# The Effect of Distance on Moral Engagement: Event Related Potentials and Alpha Power are Sensitive to Perspective in a Virtual Shooting Task

**DOI:** 10.3389/fpsyg.2015.02008

**Published:** 2016-01-07

**Authors:** Kirsten Petras, Sanne ten Oever, Bernadette M. Jansma

**Affiliations:** ^1^Department of Cognitive Neuroscience, Faculty of Psychology and Neuroscience, Maastricht UniversityMaastricht, Netherlands; ^2^Faculty of Psychology and Educational Sciences, Institute of Neuroscience, Research Institute for Psychological Science, Université Catholique de LouvainLouvain la Neuve, Belgium

**Keywords:** ERPs, moral decision making, virtual reality, avatar, EEG/ERP, EEG alpha power

## Abstract

In a shooting video game we investigated whether increased distance reduces moral conflict. We measured and analyzed the event related potential (ERP), including the N2 component, which has previously been linked to cognitive conflict from competing decision tendencies. In a modified Go/No-go task designed to trigger moral conflict participants had to shoot suddenly appearing human like avatars in a virtual reality scene. The scene was seen either from an ego perspective with targets appearing directly in front of the participant or from a bird's view, where targets were seen from above and more distant. To control for low level visual features, we added a visually identical control condition, where the instruction to “shoot” was replaced by an instruction to “detect.” ERP waveforms showed differences between the two tasks as early as in the N1 time-range, with higher N1 amplitudes for the close perspective in the “shoot” task. Additionally, we found that pre-stimulus alpha power was significantly decreased in the ego, compared to the bird's view only for the “shoot” but not for the “detect” task. In the N2 time window, we observed main amplitude effects for response (No-go > Go) and distance (ego > bird perspective) but no interaction with task type (shoot vs. detect). We argue that the pre-stimulus and N1 effects can be explained by reduced attention and arousal in the distance condition when people are instructed to “shoot.” These results indicate a reduced moral engagement for increased distance. The lack of interaction in the N2 across tasks suggests that at that time point response execution dominates. We discuss potential implications for real life shooting situations, especially considering recent developments in drone shootings which are per definition of a distant view.

## Introduction

Physical distance between an acting agent and the target of her action has long been argued to be a facilitator of moral disengagement (Milgram, 1965; Bandura, 2002; Cummings, 2006; Grossman, 2008). At present the use of remotely controlled, armed aircrafts (drones) in international conflicts imposes the question of which consequences the huge distance between pilot and target, as well as the lack of direct visual feedback, might have on the pilots' decision making. For example, while many studies have shown that killing in combat is a strong predictor for posttraumatic stress disorder (PTSD), even after controlling for direct exposure to combat as a potential confound (Fontana and Rosenheck, 1994; Maguen et al., 2010; Chappelle et al., 2011), there is some evidence suggesting that killing through a drone might not have the same impact on the drone operator: A study by the Air Force School of Aerospace Medicine in Ohio found that only 4% of drone operators screened positive for a heightened risk of posttraumatic stress disorder, while the prevalence in Iraq or Afghanistan veterans who were in close combat was estimated to range between 12 and 17% (Chappelle et al., 2011; Miller, 2012). These discoveries are in line with Milgram's findings from 1965. In a follow up to his 1963 “Obedience to authority” (Milgram, 1963) study, Milgram showed that participants obey less to an authority figure telling them to inflict pain on a victim as a punishment when the physical immediacy between the victim and the participant is increased (Milgram, 1965). The increased reluctance to hurt another person with decreased distance implies a correlation between physical proximity and obedience, which might be influenced by moral judgment.

Despite the fact that moral decision making is frequently investigated (for review see O'Fallon and Butterfield, 2005) and proximity is frequently mentioned as a potential factor in the process of moral engagement (e.g., Jones, 1991; Bandura, 2002; Paharia et al., 2009) there is little systematic research persued on the effects of perspective in moral decision making. Using a virtual reality (VR) based shooting game we aimed to investigate the interaction of perceived distance from the victim and the amount of conflict a shooter might experience. We recorded behavior as well as EEG online during the experiment. According to Milgram's findings (for review see Blass, 1999; Burger, 2009) moral conflict should be higher when the agent is physically closer to the target of a harming action. To quantify “conflict,” we looked into a shooter's neural information processing using the event related potential (ERP), as well as pre- stimulus alpha power.

Originally found by Berger (1929), alpha band activity has long been thought to reflect “cortical idling,” that is, the deactivation of currently not engaged cortical regions (Pfurtscheller et al., 1996). More recently, Jensen and Mazaheri (2010) suggested a framework in which oscillatory alpha activity serves to gate information by active inhibition of task irrelevant regions. In both views it is evident that when alpha power over occipital regions decreases, visual attention rises (Herrmann and Knight, 2001). In this manner, alpha power could serve as an indicator for task engagement and arousal (Davidson et al., 2000; Macdonald et al., 2011; VanRullen et al., 2011; Lou et al., 2014). We hypothesize that when there is a heightened moral conflict, arousal and task engagement increases, leading to a subsequent lower alpha power. Thus, ongoing alpha power should be lower with increased proximity.

An early ERP component (first negative going deflection) that has frequently been related to attentional processes is the N1 (Haider et al., 1964). The N1 component has been found to not only be sensitive to the presence or absence of a visual stimulus, but also to the vigilance of a participant asked to respond to it (Wilkinson et al., 1966). Different mechanisms have been proposed to explain the apparent coupling between a participant's task performance in visual detection paradigms and her levels of attention. Whereas, Broadbent (1958) attributed decreases in vigilance over time to extraneous stimuli directing attention away from the task, Wilkinson (1960) proposed that they are due to lowering levels of arousal. More recent research has reliably related very early components, including the N1, to valence and arousal (for review see Olofsson et al., 2008). In our paradigm, we expected N1 to reflect the state arousal of participants with respect to the different task instructions, targets and perspectives such that increased moral engagement gives rise to larger N1 amplitudes. The N1 is usually followed by a positive deflection (P2), which has been found to be sensitive to cross modal associations (Bien et al., 2012), language processes (Federmeier and Kutas, 2002), and memory (Lefebvre et al., 2005). Importantly for our paradigm, the P2 has been described to be modulated by aversive, more than by neutral stimuli (Eimer et al., 2003).

Initially, we had a particular focus on the N2. The N2 is a negative-going deflection that peaks around 200 ms post stimulus. It was initially found in monkeys performing a visually initiated hand movement task (Sasaki et al., 1989) and thought to be related to response inhibition in No-go vs. Go tasks. This initial idea has later been expanded to encompass general response conflict (Kopp et al., 1996; Donkers and van Boxtel, 2004) but the N2 has also been used as a measure for the speed of information processing (Thorpe et al., 1996; Schmitt et al., 2000). In human participants, the N2 is reported to originate from the anterior cingulate cortex (ACC) (Van Veen and Carter, 2002; Nieuwenhuis et al., 2003) which has frequently been linked to cognitive conflict (Swainson et al., 2003; Forster et al., 2010; Shenhav and Greene, 2010). The N2 component has been used as an indicator of response conflict in morally relevant decisions (Correll et al., 2006; Balconi, 2011). In Correll's study (Correll et al., 2006) participants were required to decide between virtually shooting and not shooting human targets that could be either armed or unarmed. Half of the targets were black and half of them were white. The N2 differentiated between armed/unarmed and black/white targets, having bigger amplitudes for unarmed and white targets. It seems that there was less conflict when shooting a black as opposed to a white target, indicating that participants have a prepotent response to shoot black compared to white targets. This study reveals that the N2 is sensitive to contextual alternations and can be used in addition to behavioral measures to study conflict in decision making.

In the present study, we used a modified Go/No-go task designed to quantify moral conflict across distance. Participants had to “shoot” suddenly appearing human like avatars in a virtual reality visual scene. They saw the scene either from an ego perspective with targets appearing directly in front of them or from a bird's view, where targets were seen from above and more distant. To control for low level visual features, we introduced a visually identical control condition to a second set of participants. Here, the instruction to “shoot” was replaced by an instruction to “detect” in order to abolish any moral considerations conflicting with the task. Assuming that there is more moral engagement in closer proximity, the N2 amplitude should be higher in shooting from close range, compared with shooting from the distance. At the same time, pre-stimulus alpha power should be reduced for the “ego-” compared to the “bird” condition. Both these effects should be more pronounced in combination with the task instruction that requires the participant to shoot, compared to the “detect” condition featuring visually identical stimuli, but lacking the morally aversive task instruction.

## Materials and methods

### Participants and ethics statement

Participants were 30 healthy right handed volunteers (10 male, 20 female) aged from 18 to 44 (mean = 25.96) with normal or corrected to normal vision and randomly assigned to one of two experimental conditions. They were age and gender matched between the “shoot” and the “detect” condition. In both groups, the majority of the participants were non-habitual gamers (11 in the “shoot” and 10 in the “detect” condition). Habitual gamers were defined as playing first person shooter games at least once per week or having done so within the past 2 years. Participants were naïve to the purpose of the study, and initially merely instructed to play a shooting/detection game. The study was approved by the local ethical committee of the Faculty of Psychology and Neuroscience of Maastricht University (approval number: ECP-128, 11_05_2013). All participants gave their written informed consent prior to the experiment and were informed about their right to abort the experiment at any time. Participants were fully debriefed about the purpose of the study after participation and received 10 Euros as reimbursement in the form of gift vouchers.

### Stimuli

**Figure 1:**
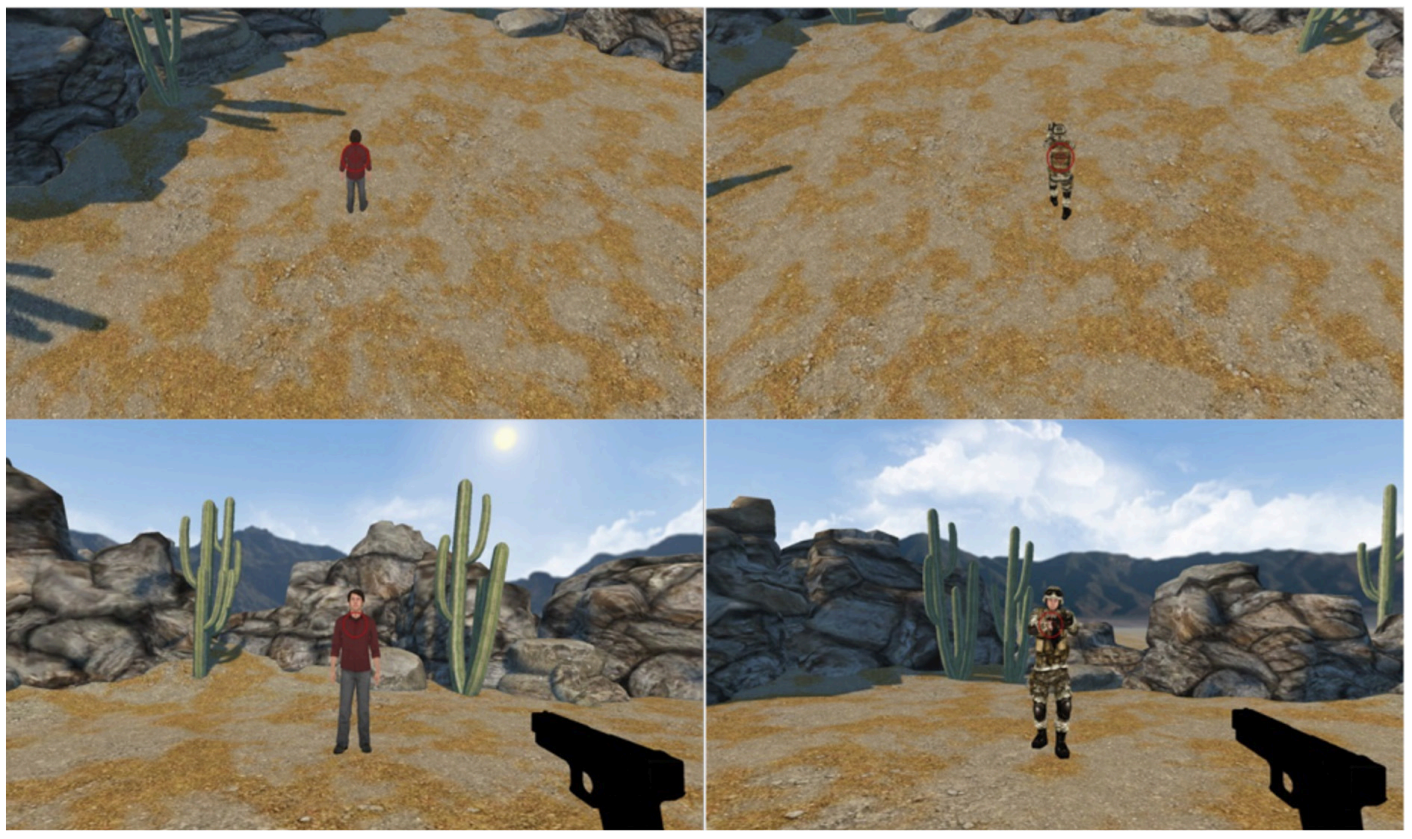
**Stimuli**. From upper left to lower right: “No-go bird,” “Go bird,” “No-go ego,” “Go ego.” Participants first saw the empty desert environment for 1–2 s until a target appeared. Targets appeared with their front/back toward the participant 50% of the time in all conditions.

The stimuli were constructed using Worldviz Vizard (Santa Barbara CA). They consisted of a virtual desert environment, presented on a computer screen, in which the participants were free to shift their view for 180° on the horizontal plane by moving a joystick with their right hand. The refresh rate of the screen was 60 Hz and the distance between participant and screen was approximately 50 cm. Within the desert display, human-like avatars appeared for 1 s, within which the participant had to either respond or refrain from responding dependent on target type. There were two types of avatars (a military soldier in uniform or a civilian in t-shirt and jeans) and two types of distances (ego or bird perspective). In the ego perspective condition the avatar was seen from a close range (approximately 2 m, observer and target are at the same ground level) while in the bird's perspective condition, the observer sees the target from above at a larger distance (see Figure 1). Note that in the ego perspective a handgun was seen in the lower right corner of the screen to make the scene more realistic. Participants were asked to shoot the soldier, not the friend (Go/No-go “shoot” instruction) or to detect the soldier, not the friend (Go/No-go “detect” instruction). Participants were different across instructions to avoid cross over effects of tasks. They were asked to trigger response with the right index finger via a button press at the game joystick. The button press was either a Go-shoot or Go-detect response. With the shooting response, a typical shooting sound was played and the participant saw a 2 s animation of the target falling down. The animation's perspective was matched to the viewing perspective of the block. In the control condition, stimuli and response types were identical to the “shoot” condition. Solely the task instruction was changed from “shoot the enemy” to “spot the man dressed in green” to avoid any moral aspects within trail and response. Further, the shooting sound as well as the falling down animation was absent.

### Experimental design and procedure

After providing written informed consent and the EEG equipment was mounted, the participants were seated comfortably in a chair in front of a computer screen inside an electrically shielded room. They had their right hand on the joystick with the index finger at the trigger button throughout the whole recording while their arm was placed on an armrest to avoid unnecessary movement.

The experiment employed a 2 × 2 × 2 between-within design in a combination of EEG measurements with virtual reality stimuli. The within subjects factors were requirement to shoot or not (Go/No-go) and perspective (bird/ego). The between subjects factor was task instruction (“shoot”/“detect”). The factor distance (Ego/Bird) was presented in four blocks (two each). Each block consisted of 100 trials, resulting in a total of 400 trials with an average duration of 3.5 s in the “shoot” condition and 2.5 s in the “detect” condition. The inter trial interval (ITI), defined as the time between one target disappearing and another one appearing was jittered to take anything between one and 2 s. Go and No-go (50% of all trials each) trials as well as back or front appearances (50% back/front each) were pseudo-randomized over blocks, allowing not more than 3 targets of the same kind (friend or enemy) to appear in direct succession to avoid possible oddball effects (Ferrari et al., 2009).

### EEG acquisition

All participants were equipped with an EEG cap (Easy Cap) holding 28 electrodes placed according to the international 10–20 system. Scalp electrodes were: F7, FT7, P7, Fp1, F3, FC3, C3, CP3, P3, O1, Fpz, AFz, Fz, FCz, Cz, CPz, Pz (ground), POz, Oz, Fp2, F4, FC4, C4, CP4, P4, O2, F8, FT8, P8. Five more electrodes were placed laterally to both lateral canthi, below the left eye to monitor for eye movements, and on the left (online reference) and right mastoid bone. The impedance was kept below 5 kΩ throughout the whole recording. EEG was recorded with a sampling rate of 250 Hz through BrainVision recorder software (BrainProducts GmbH, Munich, Germany). The signal was online referenced to the left mastoid and later offline re-referenced to the average of the left and right mastoid signal.

### Data analysis

To analyse behavioral data across “shoot” and “detect” conditions, incorrect RTs (< 4% in both conditions) were discarded from further analysis. All RTs below 200 ms or exceeding 1 s were considered incorrect as the target only remained visible for this period. Correct RTs were log transformed to account for a positive skew in the distribution and analyzed using split plot ANOVAs in a custom made Matlab-script (Matlab, Mathworks) with “task instruction” as between subject factor and “perspective” as within subject factor. Two participants were excluded from this analysis due to technical failures of recording the reaction times (one from each group).

EEG data were pre-processed using BrainVision Analyzer (BrainProducts GmbH, Munich, Germany) and the FieldTrip (Oostenveld et al., 2011) software package under Matlab, as well as custom made scripts. Data pre-processing in BrainVision Analyzer involved artifact correction using independent component analysis (ICA) to detect noisy components and inverse ICA to remove this noise from the data. On average eight components were selected. Furthermore, the data were filtered with a Butterworth zero phase filter with a low cut-off at 0.53 Hz, 12 dB/oct and a high cut-off at 20 Hz, 24 dB/oct as well as a notch filter at 50 Hz. Data were then exported to Matlab to be further analyzed. Trials were segmented from −200 to +800 ms around target onset (appearance of the avatar). The average microvolt signal per participant and condition (baseline corrected to the average of the 200 ms before stimulus onset for the ERP analysis) was subjected to split-plot ANOVAs with target (Go/No-go) and perspective (bird/ego) as repeated measures and task instruction (“shoot”/“detect”) as between subject factor. Separate ANOVAs were computed for mean amplitudes ± 50 ms around the peak of the N1, P2, N2, and P3 ERP components. Peak latency was determined as the local minimum/maximum of the grand average ERP waveform. Tested electrodes were Oz (N1 component), Fz (P2 and N2 components), and Cz (P3 component) according to visual inspection of the topographic distribution of the components. *P*-values were adjusted using the Bonferroni method to correct for multiple comparisons.

In addition to the ERP analyses, time-frequency analysis was performed in FieldTrip (Oostenveld et al., 2011) using Hanning tapers (three cycles were included for all frequencies). Data were re-epoched to 500-0 ms prior to stimulus onset and analyzed from the occipital electrodes (Oz, O1, O2, POz, P3, P4) for frequencies ranging from 2 to 30 Hz. Statistical analysis employed a split plot ANOVA with perspective as a within and task instruction as a between subject factor using the average pre-stimulus alpha power as dependent variable (between 8 and 12 Hz). For the frequency analysis, data were collapsed over Go and No-go trials since we were interested in state-dependent effects and participants could not predict the upcoming stimulus type.

## Results

### Behavioral data

The analysis of log transformed response times (Original response times: Go-ego “shoot”: *M* = 546 ms, Go-bird “shoot”: *M* = 552 ms; Go-ego “find”: *M* = 562 ms, Go-bird “find”: *M* = 560 ms) showed no significant differences for perspective [*F*_(1, 28)_ = 0.186, *p* = 0.38] or for task instruction [*F*_(1, 28)_ = 0.278, *p* = 0.33] as well as no interactions between both [*F*_(1, 28)_ = 0.625, *p* = 0.26]. False response rates were below 4% for each participant and each condition with no significant differences between conditions.

### Time-frequency analysis

**Figure 2:**
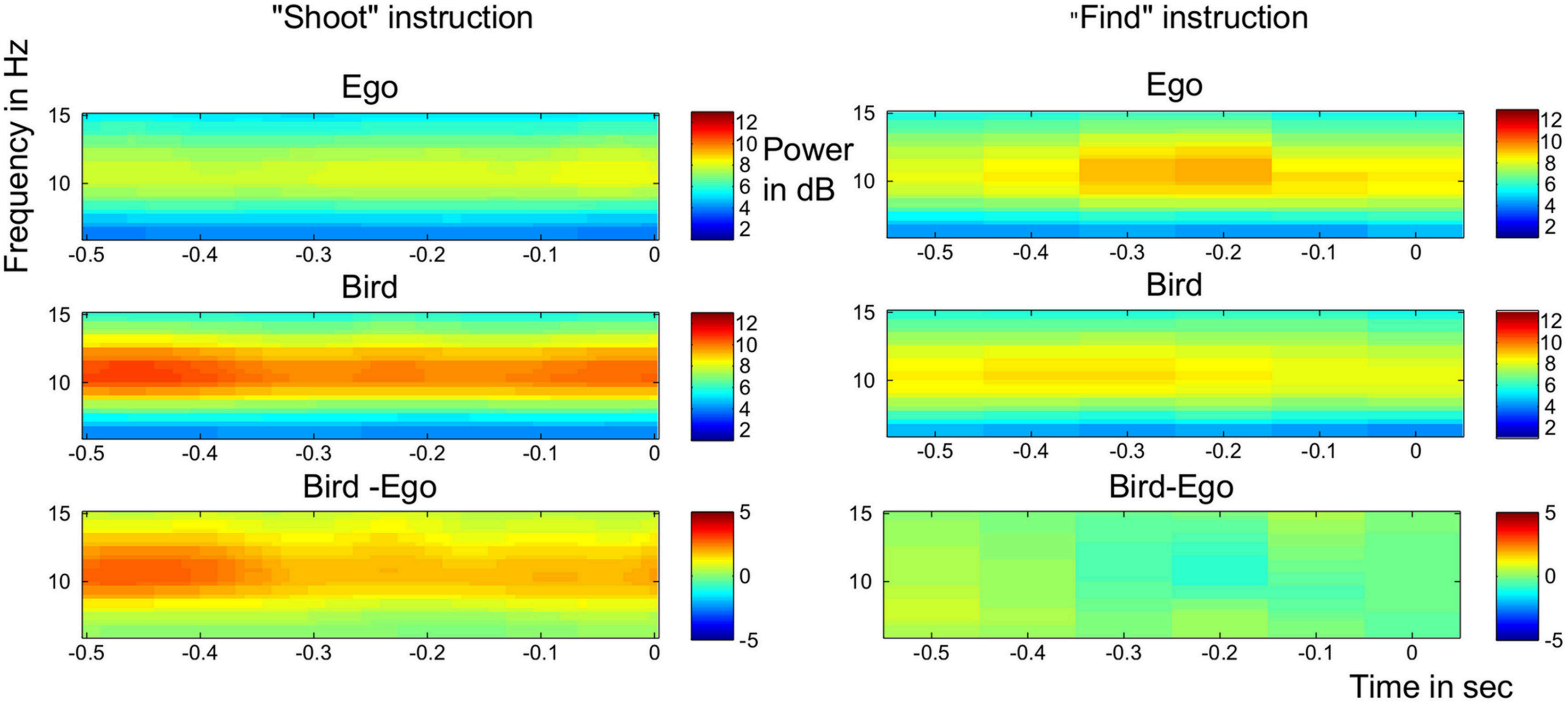
**Time-frequency analysis**. Pre-stimulus alpha power over occipital and parietal electrodes for both the “shoot” condition (left part) and the “detect” condition (right part). Alpha power was significantly reduced in the Ego condition of the “shoot” task. Upmost panel: Ego condition. Middle panel: Bird condition. Lowest panel: Contrast Bird—Ego Condition. Warm colors indicate higher alpha power.

Alpha power has been shown to be related to attention\arousal in various studies with greater alpha power indicating lower levels of arousal (Cantero et al., 1999). The split plot ANOVA revealed a significant interaction of pre-stimulus alpha power averaged over electrodes Oz, O1, O2, POz, P3, P4 between perspective and task instruction [*F*_(1, 28)_ = 4.79, *p* = 0.037] with the contrast Bird > Ego, meaning lower power in the alpha band for the ego- compared to the bird perspective, being significant only for the morally aversive “shoot”- instruction [*t*_(14)_ = −2.99, *p*_(*bonf*)_ = 0.02, partial eta squared = 0.309] but not for the neutral “find”- instruction [*t*_(14)_ = −1.16, *p*_(*bonf*)_ = 0.54, partial eta squared = 0.088] (see Figure 2). This indicates that there was more arousal for the ego compared to the bird condition, but only during the “shoot” instructions.

### ERP analysis

**Figure 3:**
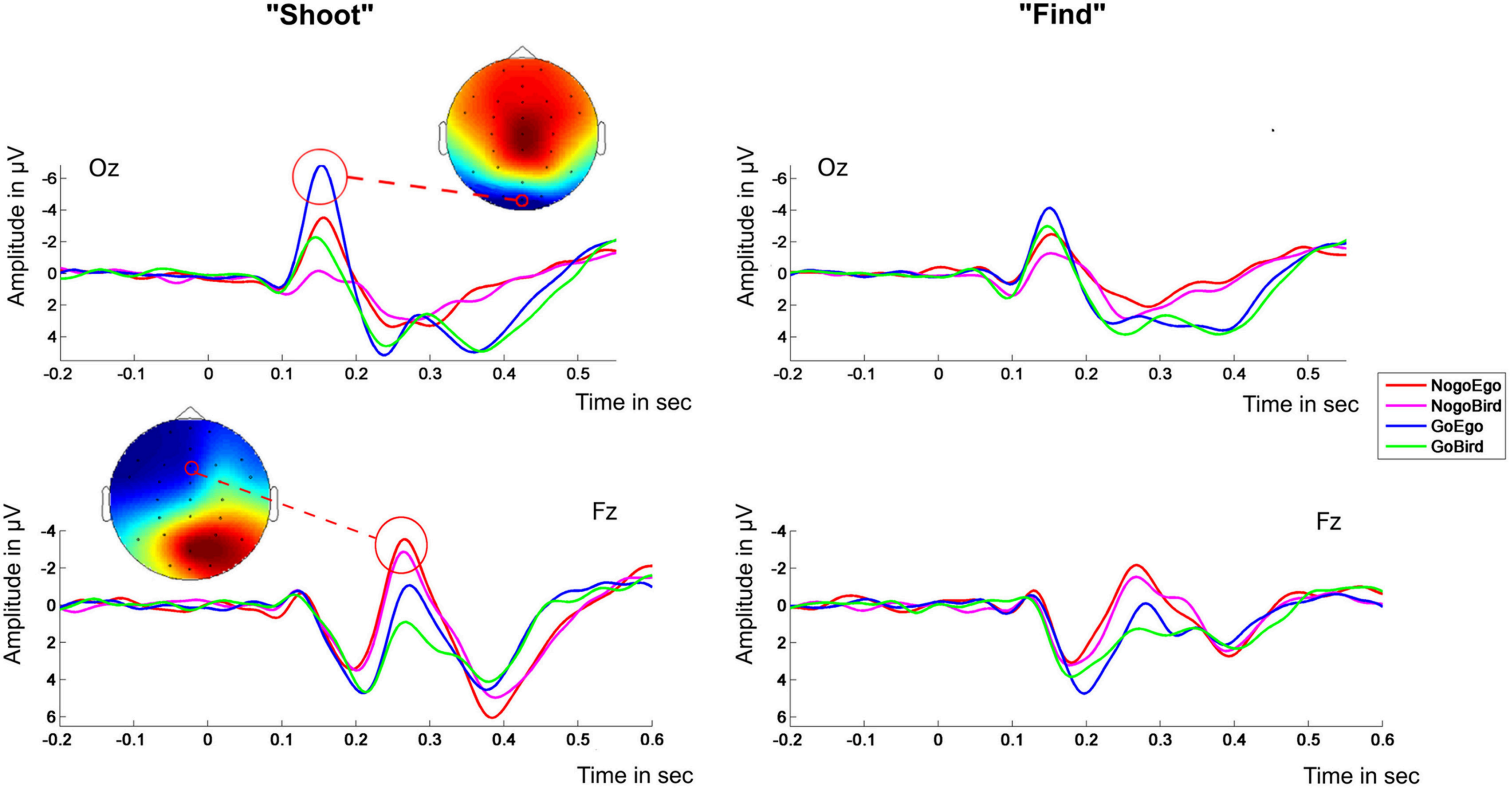
**ERP waveforms**. Left panel: “Shoot”-instruction; Right panel: “Find”-instruction. Electrodes OZ (upper half) and Fz (lower half). The scalp topographies in the left panel exemplarily show the topography of the condition eliciting the highest peak at peak time (± 25 ms). The red circle in the topography surrounds the location of the electrode of which the ERP is shown.

Visual inspection revealed a typical ERP pattern during task execution time locked to the event (appearance of an avatar) including an occipital negativity, a frontal positivity and Go/No-go negativity, and a central positivity evolving over time. The average ERP waveforms in electrodes Oz and Fz (occipital and fronto central, to demonstrate the time course of the signal for the target components) for both task instructions are displayed in Figure 3. Data from the “shoot” condition generally revealed larger amplitudes than data from the “detect” condition. Based on polarity and latency ERP components were identified as N1 (average peak latency: 152 ms), P2 (average peak latency: 204 ms), N2 (average peak latency: 272 ms), and P3 (average peak latency: 380 ms).

### N1

**Figure 4:**
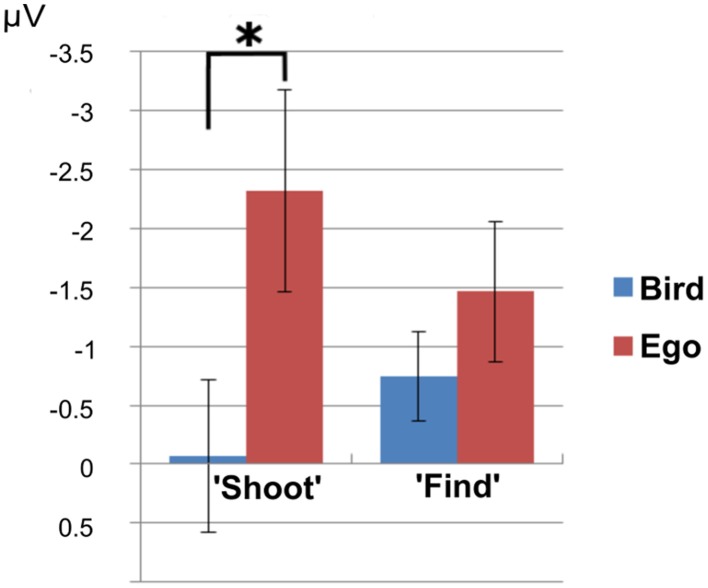
**N1 effect**. N1 amplitudes showed significantly larger amplitudes in the “Ego” compared to the “Bird”-view condition only for the “shoot”- task, but not for the “find”-task. The asterisk indicates significance at a *p*-value of 0.05.

The analysis of the N1 usually attributed to mean amplitudes in electrode Oz revealed no significant 3-way interaction between T\target, perspective and task instruction [*F*_(1, 28)_ = 4.15, *p* = 0.51]. We found a significant interaction between perspective and task instruction [*F*_(1, 28)_ = 10.59, *p* = 0.003]. Amplitudes were larger (more negative) in the “Ego” compared to the “Bird”-view condition for the “shoot”- task [*t*_(14)_ = −6.12, *p* < 0.001, partial eta squared = 0.728], but not for the “find”-task [*F*_(1, 14)_ = 5.45, *p* = 0.07] (see Figure 4). At this early component, there was no apparent interaction between target type (Go vs. No-go) and task instruction [*F*_(1, 28)_ = 4.0458, *p* = 0.054], but a significant main effect of target type [*t*_(29)_ = 4.39, *p* < 0.001, partial eta squared = 0.405]. In other words, the N1 component differentiated between perspectives, but only in the morally engaging “shoot” task. This again reflects higher arousal for the ego compared to the bird condition during the “shoot” instructions.

### P2

**Figure 5:**
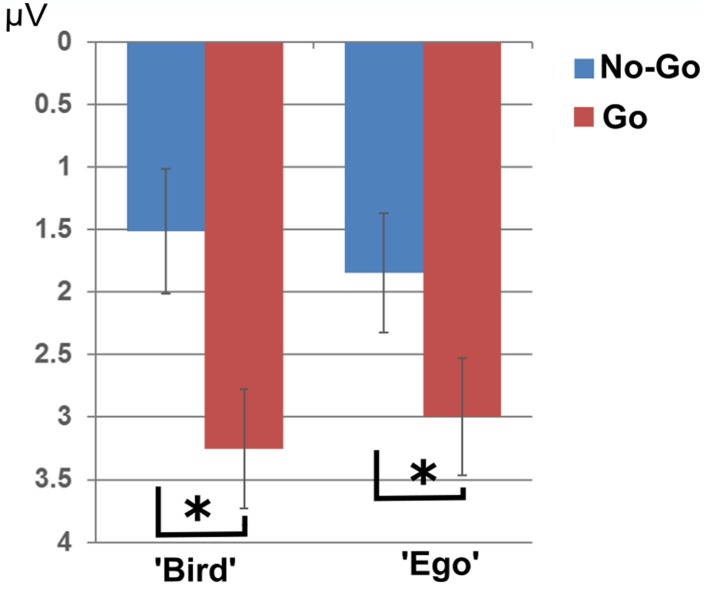
**P2 effect**. P2 amplitudes showed significantly larger amplitudes for “Go”- compared to “No-go” targets. This effect was stronger in the “Ego” compared to the “Bird” condition. The asterisks indicates significance at a *p*-value of 0.05.

The 3-way interaction between target, perspective and task instruction was not significant [*F*_(1, 28)_ = 1.31, *p* = 0.261]. The analysis revealed a main effect of target type (Go vs. No-go), as well as an interaction between target type and perspective, but did not differentiate between task instructions. Mean P2 amplitudes in electrode Fz were significantly larger for “Go”- compared to “No-go”- targets [*F*_(1, 28)_ = 28.92, *p* < 0.001]. In addition, there was a significant interaction of type of target with perspective [*F*_(1, 28)_ = 5.28, *p* = 0.03] (see Figure 5) with the contrast “No-go”—“Go” (target friend—soldier respectively) in the “ego” perspective [*t*_(29)_ = −5.68, *p* < 0.001, partial eta squared = 0.527] being more pronounced than in the “bird” view [*t*_(29)_ = −4.08, *p* < 0.001, partial eta squared = 0.364]. This indicates stronger attention for the aversive go trials compared to the no-go trials in the ego condition. However, this was independent of task instruction.

### N2

The 3-way interaction between target, perspective and task instruction did not yield significant results [*F*_(1, 28)_ = 1.52, *p* = 0.23]. Analysis of the N2 component amplitudes in electrode Fz revealed main effects for both Go/No-go target [*t*_(29)_ = −6.84, *p* < 0.001, partial eta squared = 0.619] and perspective [*t*_(29)_ = −3.41, *p* = 0.002, partial eta squared = 0.298] with higher amplitudes for the “ego”- perspective and the “No-go”- targets in both groups. There was no significant interaction between perspective and task instruction [*F*_(1, 28)_ = 1.67, *p* = 0.21].

### P3

The 3-way interaction between target, perspective and task instruction did not yield significant results [*F*_(1, 28)_ = 0.06, *p* = 0.8]. P3 amplitudes in electrode Cz differentiated between targets [*t*_(29)_ = −3.29, *p* = 0.003, partial eta squared = 0.29] with significantly larger peaks for “Go”- compared to “No-go”- targets. The main effect of perspective [*F*_(1, 28)_ = 1.53, *p* = 0.23] and the interaction between perspective and group [*F*_(1, 28)_ = 0.75, *p* = 0.39] were not significant.

## Discussion

The objective of the current study was to investigate whether the amount of conflict associated with a moral violation (to kill) in a virtual shooting task depends on the perceived distance between shooter and target. Our aim was (1) to quantify task dependent arousal by modulation in alpha power, with higher levels of arousal reflected by decreased alpha power and (2) to quantify conflict by means of the N2 amplitude, with higher amplitudes reflecting more conflict.

Pre-stimulus alpha power showed the expected task and perspective dependent modulations with higher power, signifying less engagement, for the “bird” than the “ego” perceptive, but only in the morally engaging “shoot” task. These results indicate that although the two conditions where visually identical during the analyzed time intervals the different task instructions changed the overall information processing. Further, as expected, we observed higher N2 amplitudes for the “ego” compared to the “bird” perspective and for the “No-go” compared to “Go” targets—demonstrating successful application of ERP in complex VR like experimental settings. Interestingly, we did not find a significant interaction between perspective and task instruction in the N2 amplitudes. Instead, we found this interaction in the N1 amplitudes.

### Behavioral effects

We did not observe any significant effects of task instruction or distance on reaction times or accuracies. This is likely the case because task requirements (respond to one of two targets which occurred with equal likelihood) were identical across conditions and could theoretically be resolved by a simple color discrimination. Although the color discrimination for the distant condition was marginally more difficult due to a smaller target size, this deviation was not sufficient to confound reaction times or accuracies.

### Alpha and N1 effects

Alpha power 500 ms prior to stimulus onset was generally lower in the “shoot,” compared to the “detect” condition and differentiated between perspective conditions only in the “shoot” condition, with lower alpha power for the ego—compared to the bird perspective. This is in line with our hypothesis that intensified moral engagement causes an elevation of arousal and therefore a lower alpha power. While the two conditions were visually identical during the analyzed time intervals, they differed from each other in only two aspects: (1) whereas in the “shoot” condition, there was a shooting sound and an animation of the target falling down subsequent to all Go responses, these features were missing in the “detect” condition to not make the idea of shooting obvious to the participants. (2) The task instruction was altered from “Shoot the enemy; do not shoot the friend” in the “shoot” condition to “Find the person dressed in green” in the “detect” condition.

Both aspects could have contributed to higher arousal. The post-response differences likely have altered the participants' cognitive state toward a greater expectancy of aversive stimuli in the “shoot” task, where the animation of the target falling down was seen either from close range or from the distance, depending on perspective condition. An abundance of evidence suggests that attention and arousal have a crucial influence on alpha power (Shaw, 1996; Cantero et al., 1999; Keil et al., 2001; Simons et al., 2003). Likewise, the wording of the task instruction might have diminished the morally aversive content of the task and influenced pre-stimulus alpha power. According to Bandura, one way to disengage moral agency is the use of “sanitizing language” (Bandura, 1999). In this framework, the “find” instruction in the “detect” condition could be perceived as a euphemism for shooting, lowering attention and arousal levels induced by the moral context and therewith lowering moral engagement.

To which extend the post stimulus, post response visual features and the wording of the task instruction respectively influenced the alpha power needs to be addressed in subsequent research. However, the moral context and the participant's state seem to modulate stimulus processing from early on. Further support for this hypothesis comes from the N1 results. N1 amplitude has been related to increased attention and arousal (Eason et al., 1969; Hillyard and Münte, 1984; Hillyard et al., 1995; Hillyard and Anllo-Vento, 1998; Carretié et al., 2004). More recently it has been shown that N1 next to arousal and attention is also sensitive to cross modal integration, and meaning (Nager et al., 2006; Sinke et al., 2014). In the current experiment, N1 amplitudes showed significant differences dependent on perspective only in the “shoot” condition, but not in the “detect” condition. Together, these results indicate higher levels of state arousal (and by that an influence of moral state) on the early N1 component for the “shoot” instruction in general and for the ego condition in the “shoot” instruction in particular. Thus, it seems like the differences in moral context between instructions and conditions altered moral engagement.

### N2 effects

The significant main effects for perspective on the N2 amplitudes indicate that distance, although irrelevant for the task, has a crucial influence on the N2 component. This finding is in line with our hypothesis that proximity to the effects of a self-induced harming action results in enhanced conflict which in turn is reflected in the amplitude of the N2 ERP component. However, we did not observe an interaction between perspective and task “shoot” vs. “detect.” One explanation might be that the display put most of them into a “shooting” mode. This was confirmed by post hoc interviews of participants. When asked after the EEG session what they thought they were doing during the experiment, all participants, independent from which of the two task instructions they had received, reported, that they were “shooting” the “Go” target. Therefore, the implicit task understanding was similar in both conditions and we were not able to effectively control for the moral content of the task.

Further, due to the lack of task interaction, we cannot directly exclude that the visual differences (scene complexity higher in bird view) and/or differences in task difficulties (target detection more difficult in bird view because the target was displayed smaller) between the two perspectives guides the N2 effect. But we consider this as unlikely for several reasons: Preceding N2 studies related the N2 to task difficulties and observed higher N2 amplitudes with higher visual or task complexity (Senkowski and Herrmann, 2002). We however observed the opposite pattern. The N2 amplitudes in the present study were consistently more pronounced in response to the less difficult visual discrimination (targets appear bigger in the ego perspective). Also general task difficulty can be ruled out as cause for N2 modulations. The task difficulty as reflected by reaction times did not differ across conditions.

Moreover, the N2 component could have been sensitive to aversive stimuli (Dennis and Chen, 2009), showing higher amplitudes to threatening than to neutral stimuli. In contrast, here the N2 amplitudes were consistently higher for the non-threatening “friend” compared to the potentially threatening soldier. Also the differences in the P2, preceding the N2 component do not explain the variations in the N2: The P2 showed significantly larger amplitudes for Go- compared to No-go stimuli. Considering that the P2 component has been found to be sensitive to threatening stimuli (Eimer et al., 2003) and the “Go”-targets were avatars with a military uniform, this effect was expected. Nevertheless, P2 amplitudes failed to show significant differences for the perspective, suggesting a clear functional segregation of neural processing of threat (P2) and distance/conflict (N2).

Altogether, the N2 results cannot easily be explained without assuming some sort of conflict involvement. Given the link between N2 amplitudes in EEG and ACC activation in fMRI (Van Veen and Carter, 2002) our interpretation finds additional support by an fMRI study (Greene et al., 2004). Greene and colleagues reported increased activation of conflict sensitive ACC upon decisions requiring personal moral violations. Note however that there is an on-going debate about the details of the cognitive processes underlying ACC activation. Some refer to it as “conflict monitoring,” some as “cognitive control” (Bechara et al., 1999; Dolan, 2002; Bechara, 2004; Botvinick, 2007). Thus, the alpha and N1 results show an increased moral engagement for the “shoot” task, especially in the ego condition. However, our N2 results do not unambiguously reflect a parallel amount of moral conflict. Although one would expect that heightened moral engagement increases the amount of moral conflict, our result do not seem to directly support this.

Note however, that insufficient statistical power given our sample size of 30 participants in total (15 per task instruction) might have limited the significance of some of the statistical comparisons. A *post-hoc* power analysis using G^*^power (Faul et al., 2009) revealed that a sample size of 31 participants per task instruction would have been required to detect the observed effect size (partial eta squared = 0.056) at the recommended 0.80 level (Cohen, 1988) for the interaction between task instruction and perspective.

## Conclusion

In summary, we found that increased distance toward avatars in VR based shootings modulates the N1, P2, N2, and alpha power during task performance. The paralleling significant differences in the N1 time-range and the pre-stimulus alpha power observed in the “shoot” condition for the two different perspectives suggest decreased attention and arousal with increased distance. These results validate theoretical accounts that increased distance to a target leads to a decrease in moral engagement. Although further studies with more suitable controls are needed to verify the role of N2 in this paradigm, our alpha and N1 results have potential implications for real life shooting situations, conflict coping, and PTSD treatments. This type of research is specifically applicable to measure conflict in sniper shootings or even during the use of remote killing techniques such as armed drones.

## Author contributions

KP designed and conducted the experiment, analyzed the data and wrote the manuscript. ST contributed to designing and conducting the experiment, analyzing the data and writing and revising the manuscript. BJ supervised experimental design and data collection and contributed to data analysis as well as writing and revising the manuscript.

### Conflict of interest statement

The authors declare that the research was conducted in the absence of any commercial or financial relationships that could be construed as a potential conflict of interest.
